# Genome-wide member identification, phylogeny and expression analysis of PEBP gene family in wheat and its progenitors

**DOI:** 10.7717/peerj.10483

**Published:** 2020-12-15

**Authors:** Lei Dong, Yue Lu, Shubing Liu

**Affiliations:** State Key Laboratory of Crop Biology, College of Agronomy, Shandong Agricultural University, Tai’an, China

**Keywords:** Wheat, *PEBP*, Gene family, Phylogeny, Expression profile

## Abstract

The phosphatidylethanolamine binding protein (PEBP) family comprises ancient proteins found throughout the biosphere that play an important role in plant growth and development, flowering, seed development and dormancy. However, not all *PEBP* genes have been identified or analyzed in common wheat (*Triticum aestivum* L.) and its progenitors. In this study, we identified the *PEBP* genes in common wheat, *Triticum dicoccoides*, *Triticum urartu* and *Aegilops tauschii* by searching whole genome sequences, and characterized these genes by phylogenetic and transcriptome analyses. A total of 76, 38, 16 and 22 *PEBP* genes were identified in common wheat, *T. dicoccoides*,* T. urartu* and *Ae. tauschii*, respectively. Phylogenetic analysis classified the *PEBP* genes into four subfamilies (*PEBP-like*, *MFT-like*, *TFL-like* and *FT-like*); the *PEBP-like* subfamily was identified as a new subfamily with genes in this subfamily were conserved in plants. Group 2, 3 and 5 chromosomes of common wheat and its progenitors contained more *PEBP* genes than other chromosomes. The *PEBP* genes were conserved in wheat during evolution, and tandem duplication played a more important role in the amplification of *PEBP* genes than segmental duplication. Furthermore, transcriptome analysis revealed that *PEBP* genes showed tissue/organ-specific expression profiles and some *PEBP* genes were induced to express by biotic stresses. Quantitative real-time PCR (qRT-PCR) analysis revealed that seven randomly selected *PEBP* genes expressed differently during seed germination under cold, drought, flood, heat and salt stress treatments, and five of these genes (*TaPEBP1*, *TaPEBP5*, *TaPEBP9*, *TaPEBP66* and *TaPEBP69*) showed significantly higher expression under different stress treatments, indicating that these genes play important roles during seed germination under stress conditions.

## Introduction

The phosphatidylethanolamine binding protein (PEBP) family comprises highly conserved proteins and is represented in all three major phylogenetic divisions: Eukaryota, Bacteria and Archaea (Banfield et al., 1998; Chautard et al., 2004; Hengst et al., 2001). In *Arabidopsis thaliana*, six *PEBP* genes have been identified and grouped into three subfamilies: *TFL1-like*, *FT-like* and *MFT-like* (Kardailsky et al., 1999). The *TFL-like* subfamily comprises three genes, *TERMINAL FLOWER1* (*TFL1*), *ARABIDOPSIS THALIANA CENTRORADIALIS* (*ATC*) and *BROTHER OF FT AND TFL1* (*BFT*); the *FT-like* subfamily contains two genes, *FLOWERING LOCUS T* (*FT*) and *TWIN SISTER OF FT* (*TSF*); the *MFT-like* subfamily contains only one gene, *MOTHER OF FT AND TFL1* (*MFT*). The MFT-like subfamily is ancestral to the FT-like and TFL1-like subfamilies. The MFT-like proteins function in the gametophyte, sporophyte, and seed development and germination of bryophytes, whereas the FT-like and TFL1-like proteins play important roles in the vegetative-to-reproductive phase transition in seed plants (Liu et al., 2016).

The plant *PEBP* genes including Arabidopsis *TFL1* and tomato (*Solanum lycopersicum*) *SELF PRUNING* (*SP*) were initially cloned from mutants with altered inflorescence structure (Bradley et al., 1997; Pnueli et al., 1998). The *TFL1* gene controls both vegetative and reproductive phase durations by maintaining both apical and inflorescence meristem indeterminacy (Bradley et al., 1997; Shannon & Meeks-Wagner, 1991). Mutation of the tomato *SP* gene changed the uncertain growth habit to a definite type, resulting in bud growth restriction and dense compact habit (Pnueli et al., 1998). These mutant phenotypes indicated that *TFL1* and *SP* genes maintain the uncertain state of inflorescence meristem. The expression of *TFL1* and its paralog, *ATC*, was weak in the inner cells of mature shoot meristem during the vegetative phase but increased following the transition to the reproductive phase (Bradley et al., 1997; Ratcliffe, Bradley & Coen, 1999). The *BFT* gene (*TFL1-like* subfamily) plays an important role in the growth of plant meristem (Mimida et al., 2001). The *FT* gene demonstrates the opposite function of *TFL1* in Arabidopsis, promoting the vegetative-to-reproductive phase transition (Gursky et al., 2018; Taoka et al., 2011). FT is a key activator of flowering that mediates both photoperiod and vernalization regulation. FT and BFT may provide an adaptation strategy that finetunes photoperiodic flowering under high salt stress (Ryu et al., 2014). The function of *FT* is also highly conserved in plants. The tomato *FT* homolog, *SINGLE FLOWER TRUSS* (*SFT*), also regulates flowering time and shoot architecture by generating a graft-transmissible signal. TSF is also a floral activator, and mutation of the *TSF* gene delays flowering (Kardailsky et al., 1999; Yamaguchi et al., 2005). The *FT* and *TSF* genes are up-regulated in chaperone cells of phloem under long periods of sunshine (Jang, Torti & Coupland, 2009). MFT regulates dormancy through the mediation of gibberellin (GA) and abscisic acid (ABA) (Xi et al., 2010). In germinated seeds, the expression of *MFT* is directly regulated by transcription factors ABA-INSENSITIVE3 (ABI3) and ABI5 in the ABA signaling pathway. In addition, MFT promotes embryo growth by directly inhibiting ABI5 through a negative feedback loop (Xi et al., 2010). These results indicate that the plant PEBP family members play an important role in the vegetative-to-reproductive phase transition and seed dormancy (Bradley et al., 1997; Kojima et al., 2002; Xi et al., 2010).

Some of the *PEBP* genes have been studied in the Gramineae family. In rice (*Oryza sativa* L.), the FT protein encoded by the *Heading date 3a* (*Hd3a*) gene, also known as *OsFTL2*, migrates from leaves to meristem tips to induce flowering (Kojima et al., 2002). In wheat and barley (*Hordeum vulgare* L.), the *VERNALIZATION LOCUS3* (*VRN3*) gene, an ortholog of the *FT* gene, also plays an important role in flowering regulation (Faure et al., 2007; Yan et al., 2006). The *MFT* homolog, *TaPHS1*, of wheat regulates grain dormancy (Liu et al., 2013). Compared with the study of individual *PEBP* genes, the identification of the *PEBP* gene family is equally important in plants but relatively less common (Zhao et al., 2020). For example, 23 *PEBP* genes have been identified in soybean (*Glycine max* L.), which is almost 4-fold higher than the number of *PEBP* genes in Arabidopsis. Among these genes, *GmFT2a* and *GmFT5a* control flowering by regulating the photoperiod pathway (Wang et al., 2015; Nan et al., 2014). In maize (*Zea mays* L.), 24 *PEBP* genes have been identified, of which only *ZCN8* shows the most similar function to the *Arabidopsis FT* gene (Meng, Muszynski & Danilevskaya, 2011). Chardon & Damerval (2005) used expressed sequence tag (EST) and genomic sequence databases to carry out phylogenetic analyses of the Gramineae *PEBP* genes, and identified a total of 19, 9, and 10 *PEBP* genes in wheat, barley and rice, respectively (Chardon & Damerval, 2005). Halliwell et al. (2016) identified 12, 12, 13, and 12 *FT-like* genes in wheat, barley, rice and Brachypodium (*Brachypodium distachyon*), respectively (Halliwell et al., 2016).

Common wheat is one of the most important crops in the world, accounting for more than 50% of the total human food consumption (Shewry & Hey, 2015). Recent genome sequencing of common wheat and its progenitors allows detailed analysis of *PEBP* gene families in these species (Appels et al., 2018; Avni et al., 2017; Ling et al., 2018; Luo et al., 2017). In this study, we aimed to identify the gene structure, physical properties, chromosomal location of *PEBP* genes and their phylogenetic relationship in common wheat and its progenitors. We searched the *PEBP* genes in common wheat and its progenitors, and analyzed their expression patterns in various tissues, during the process of grain development and under various biotic and abiotic stresses. We also examined the expression of seven *TaPEBP* genes under adverse germination conditions. The results of this study provided valuable information for further investigation of the evolution and molecular function of *PEBP* genes in common wheat and its progenitors.

## Materials & Methods

### Identification of *PEBP* family members

The complete protein sequences of the common wheat cultivar Chinese Spring (*Triticum aestivum* L., AABBDD, 2*n* = 6*x* = 42, IWGSC RefSeq_v1.0 & v2.0), wild emmer cultivar Zavitan (*Triticum dicoccoides*, AABB, 2*n* = 4*x* = 28, WEWSeq_v1.0 & v2.0) and *Aegilops tauschii* cultivar AL8/78 (DD, 2*n* = 2*x* = 14, AET_v4.0) were downloaded from the Ensembl database (http://plants.ensembl.org/index.html). Protein sequences of *Triticum urartu* G1812 (AA, 2*n* = 2*x* = 14, G1812 Tu_2.0) was downloaded from the MBKBASE website (http://www.mbkbase.org/Tu/). To identify *PEBP* gene candidates, protein sequences of common wheat and its progenitors were searched using two methods. In the first method, six *PEBP* genes of Arabidopsis were used to search the protein database of common wheat and its progenitors by blastp (e-value ≤ 1e^−5^). The second method was to obtain the Hidden Markov model (HMM) of PEBP (PF01161) from Pfam website (http://pfam.xfam.org/) and use it to retrieve all protein databases. All of the searched putative PEBP proteins were submitted to the CDD (https://www.ncbi.nlm.nih.gov/Structure/cdd/wrpsb.cgi) and SMART (http://smart.embl-heidelberg.de/) databases to confirm the conserved PEBP domain. The second method was also used to identify the novel PEBP-like subfamily in monocotyledonous plants including barley, *Oryza sativa* ssp. *japonica*, sorghum (*Sorghum bicolor* L.), maize and Brachypodium, and dicotyledonous plants including Arabidopsis, cotton (*Gossypium raimondii*), soybean (*Glycine max*), tomato (*Solanum lycopersicum*), cucumber (*Cucumis sativus*) and tobacco (*Nicotiana attenuata*) in the Ensembl Plants database. The predicted protein sequences lacking the PEBP domain were excluded from the analysis. Each PEBP gene must contain a complete PEBP conserved domain. When there are multiple transcripts of this gene, the most consistent transcripts (transcripts with the lowest e-value) with the HMM model are selected as PEBP genes. These *PEBP* genes in *T. aestivum* L., *T. dicoccoides*, *T. urartu* and *Ae. tauschii* were designated as *TdPEBP*, *TuPEBP* and *AetPEBP* genes, respectively, and the *PEBP* genes in Arabidopsis were designated as *AtPEBP.* For each species, the PEPB genes were numbered according to the sequence of homologous groups (A, B and D) and the physical location from small to large on the chromosomes (Ouyang et al., 2009).

### Phylogenetic analysis and classification of *PEBP* genes

Full-length amino acid sequences of the PEBP proteins from Arabidopsis as well as common wheat and its progenitors were used for phylogenetic analysis. Phylogenetic relationship was inferred using the maximum likelihood (ML) method, based on the LG model, with MEGA7.0 software (Kumar, Stecher & Tamura, 2016). A midpoint rooted base tree was produced using the Evolview (https://www.evolgenius.info/evolview/). The *PEBP* genes of common wheat and its progenitors were divided into different groups, according to the topological structure of phylogenetic tree and clustering of Arabidopsis *PEBP* genes.

### Sequence analysis and structural characterization

All highly reliable PEBP sequences were submitted to ExPASy (http://web.expasy.org/protparam/) to calculate the number of amino acids and to determine their isoelectric point (pI), molecular weight (MW) and instability index. The subcellular localization of PEBPs was predicted using CELLO (http://cello.life.nctu.edu.tw/). The chromosomal location and exon number of all *PEBPs* were obtained from the Triticeae Multi-omics Center (http://wheatomics.info/). Conserved *PEBP* sequences were identified with the MEME 5.0 suite (http://meme-suite.org/) using parameters established for Arabidopsis PEBP protein sequences, and conserved PEBPs were identified based on the following criteria: each sequence comprised non-overlapping repeats of each motif >1; number of different motifs = 20; motif width = 6–50 amino acids (aa). The predicted motifs were visualized using the TBtools software (https://github.com/CJ-Chen/TBtools). The annotation information of *PEBPs* was interpreted using GSDS version 2.0 (http://gsds.gao-lab.org/) to determine the gene structure and intron/exon distribution (Hu et al., 2015).

### Chromosomal location and tandem duplication

The chromosomal location of *PEBP* genes was obtained from the Triticeae Multi-omics Center. MapChart was used to draw the map of the chromosomes harboring the *PEBP* genes and to indicate the relative distance between two *PEBP* genes on the same chromosome (Voorrips, 2002). Tandem repeats of the *PEBP* genes were confirmed based on two criteria: (a) aligned length of shorter sequences covering the longer sequences >70%; (b) similarity between two aligned sequences >70% (Gu et al., 2002; Yang et al., 2008). Two genes on the same chromosome within a 100-kb physical distance were designated as tandem repeats (Wang et al., 2010). Synonymous substitution (Ks) and non-synonymous substitution (Ka) rates of tandem repeats were calculated as described previously (Wang et al., 2010). The divergence time (*T*) is obtained using the synonym for 6.5 × 10^−9^ per substitution per year, i.e., *T* = Ks / (2 × 6.5 × 10^−9^) (Gaut et al., 1996; Lynch & Conery, 2000; Wolfe, Sharp & Li, 1989). The number of tandem duplication events of gene families and the collinearity between wheat and other species were performed using the MCScanX software (Wang, Li & Paterson, 2013). A wheat PEBP gene, which has no collinear relationship with wheat progenitor species, is considered to be a wheat specific PEBP gene. Segmental duplications of *TaPEBP* genes were identified using the Circos software (Krzywinski et al., 2009).

### Expression analysis of *TaPEBP* genes

To analyze the expression of *TaPEBP* genes, the RNA-seq data of different tissues at adult stage including leaf, root, spike, stem and grain (at 10, 20 and 30 days post anthesis [DPA]) of Chinese Spring was downloaded from the expVIP website (http://www.wheat-expression.com/) (Borrill, Ramirez-Gonzalez & Uauy, 2016). Additionally, RNA-seq data of plants under different biotic stresses (powdery mildew and stripe rust) and abiotic stresses (cold, drought, heat and salt) were downloaded from the Triticeae Multi-omics Center. Heat maps were generated using the TBtools software. Hierarchical cluster analysis was conducted based on log2 of the transcripts per million (TPM) values of *TaPEBP* genes.

### Plant material and abiotic stress treatments

The expression of *TaPEBP* genes was verified using the wheat variety SN5058. Seeds were sterilized with 75% ethanol and then absorbed in an incubator at 25 °C for 8 h. To perform drought, flood, salt, cold and heat stress treatments, the sterilized seeds were exposed to 20% PEG6000, 10-mL sterile water, 200 mM NaCl, 4 °C and 45 °C, respectively. Three biological replicates were performed for each treatment, and each replicate contained 10 embryos. To analyze gene expression, embryos were collected at 0, 3, 6 and 12 h, immediately frozen in a liquid nitrogen tank and stored at −80 °C for RNA extraction.

### Quantitative real-time PCR (qRT-PCR) and data analysis

Expression profiles of seven randomly selected *TaPEBP* genes, including three that showed differential expression in the embryo, endosperm and seed coat (*TaPEBP1*, *TaPEBP5* and *TaPEBP66* [We use differential expression index greater than 5 to determine differentially expressed genes. The differences of *TaPEBP1*, *TaPEBP5* and *TaPEBP66* were 8.8, 16.8 and 8.6 times, respectively.]) and four that showed no differential expression (*TaPEBP9*, *TaPEBP28*, *TaPEBP50* and *TaPEBP69*), were verified by qRT-PCR. Total RNA was extracted using the RNAsimple Total RNA Kit (TransGen Biotech, Beijing, China) and reverse transcribed using the PrimeScript RT Reagent Kit with gDNA Eraser (TaKaRa, Beijing, China), according to the manufacturer’s instructions. Then, qRT-PCR was performed using the TransStart Top Green qPCR SuperMix (TransGen Biotech, Beijing, China) on the Light Cycler 96 Detection System (Roche, Switzerland) in a 20- µL reaction volume containing 10 µL of 2X Green Mix, 1 µL of each primer (10 µM), 1 µL of cDNA template (∼400 ng/ µL) and 7 µL of double distilled water. The PCR conditions were as follows: pre-denaturation at 94 °C for 3 min, followed by 40 cycles of denaturation at 94 °C for 10 s, annealing/extension and collection of fluorescence signal at 60 °C for 30 s. Three replications were performed for each cDNA. The *Actin* gene was used as an endogenous control, and gene-specific primers were designed using Primer Premier 5.0 (Table S1). Relative gene expression levels were determined using the 2^−ΔΔ*Ct*^ method. The expression level of *TaPEBP* genes was plotted using the TBtools software. Statistically significant differences between the control and treatment groups were calculated using the independent Student’s *t*-test.

## Results

### Identification of *PEBP* genes

A total of 76, 38, 16, 22 and 7 *PEBP* genes were identified in common wheat, *T. dicoccoides*, *T. urartu*, *Ae. tauschii* and Arabidopsis, respectively. In addition, the identified *TaPEBP* genes and *TdPEBP* genes were corrected with the newly released and updated genome sequences, IWGSC RefSeq_v2.0 (no gff3 file) and WEWSeq_v2.0 (no gff3 file), respectively, and the chromosome positions of four *TaPEBP* and two *TdPEBP* genes, not mapped to any chromosome in the previous genome sequence assemblies, were determined (Table S2). Among the 152 *PEBPs* in common wheat and its progenitors, *TaPEBP23* and *AetPEBP4* were identified as genes encoding the smallest proteins with 152 aa, whereas *AetPEBP1* encoded the largest protein (245 aa). The MW of the encoded PEBP proteins ranged from 16.93 to 26.91 kDa, and the pI ranged from 4.84 (*TaPEBP1* and *TaPEBP5*) to 9.85 (*TaPEBP73*). The predicted subcellular localization indicated that the PEBP proteins were located in the cytoplasm (41.9%), nucleus (6.6%), mitochondria (8.7%), chloroplast (5.8%), plasma membrane (2.1%) and extracellular space (34.9%).

### Phylogenetic analysis and classification of *PEBP* genes

**Figure 1:**
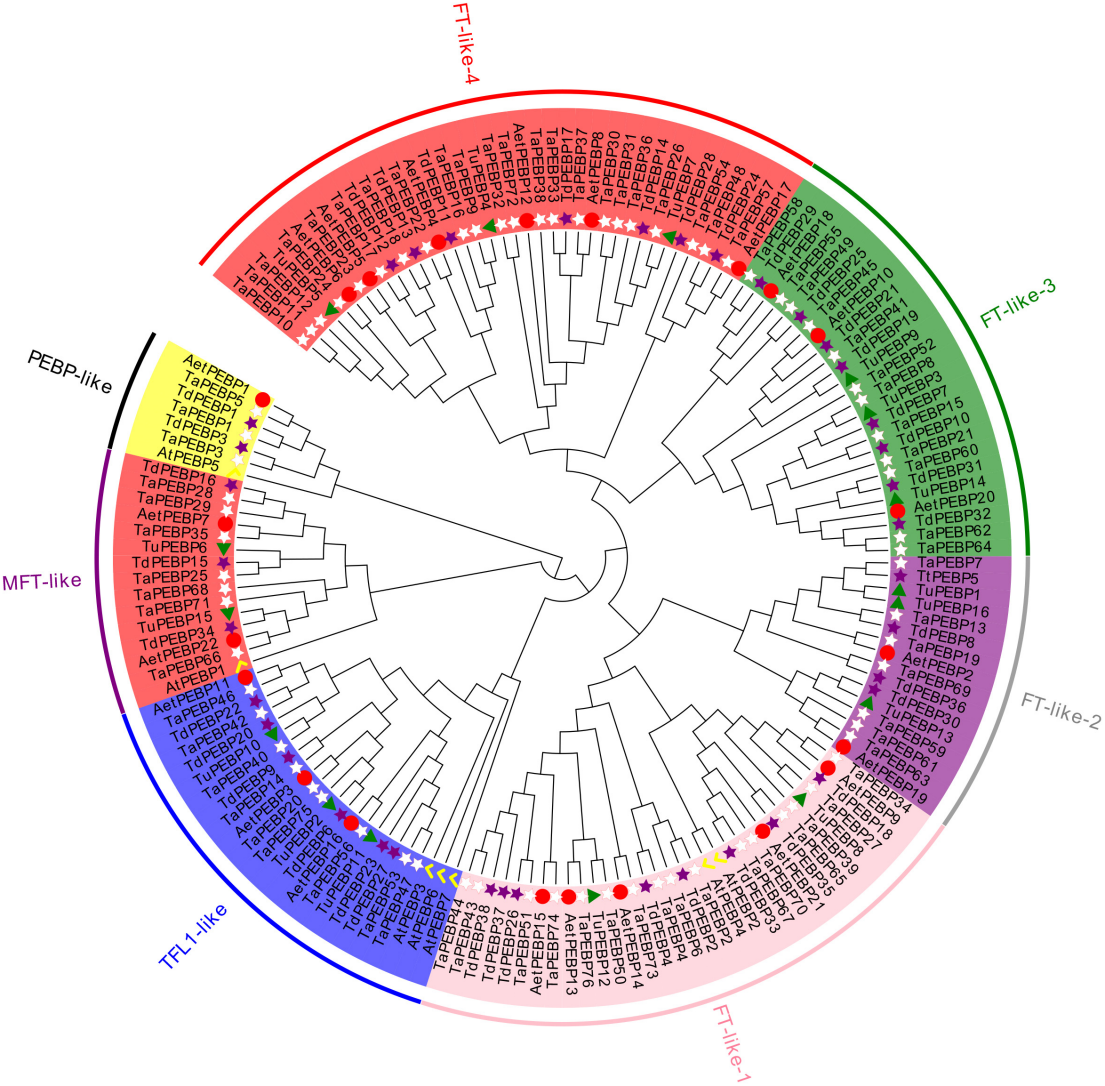
Phylogenetic analysis of *PEBP* genes in *Triticum aestivum*, *T. dicoccoides*, *T. urartu*, *Ae. tauschii* and* Arabidopsis thaliana*. The phylogenetic tree depicts the relationships among 76 *TaPEBP* (white star), 38 *TdPEBP* (purple star), 16 *TuPEBP* (green triangle), 22 *AetPEBP* (red circle) and 7 *AtPEBP* (yellow check)** genes. The rootless tree was constructed using the LG model of MEGA7.0 and divided into four subfamilies, of which the *FT-like* subfamily was further divided into four classes. Different groups are marked with different branch color branches and the same background color to indicate various *PEBP* gene types.

**Figure 2:**
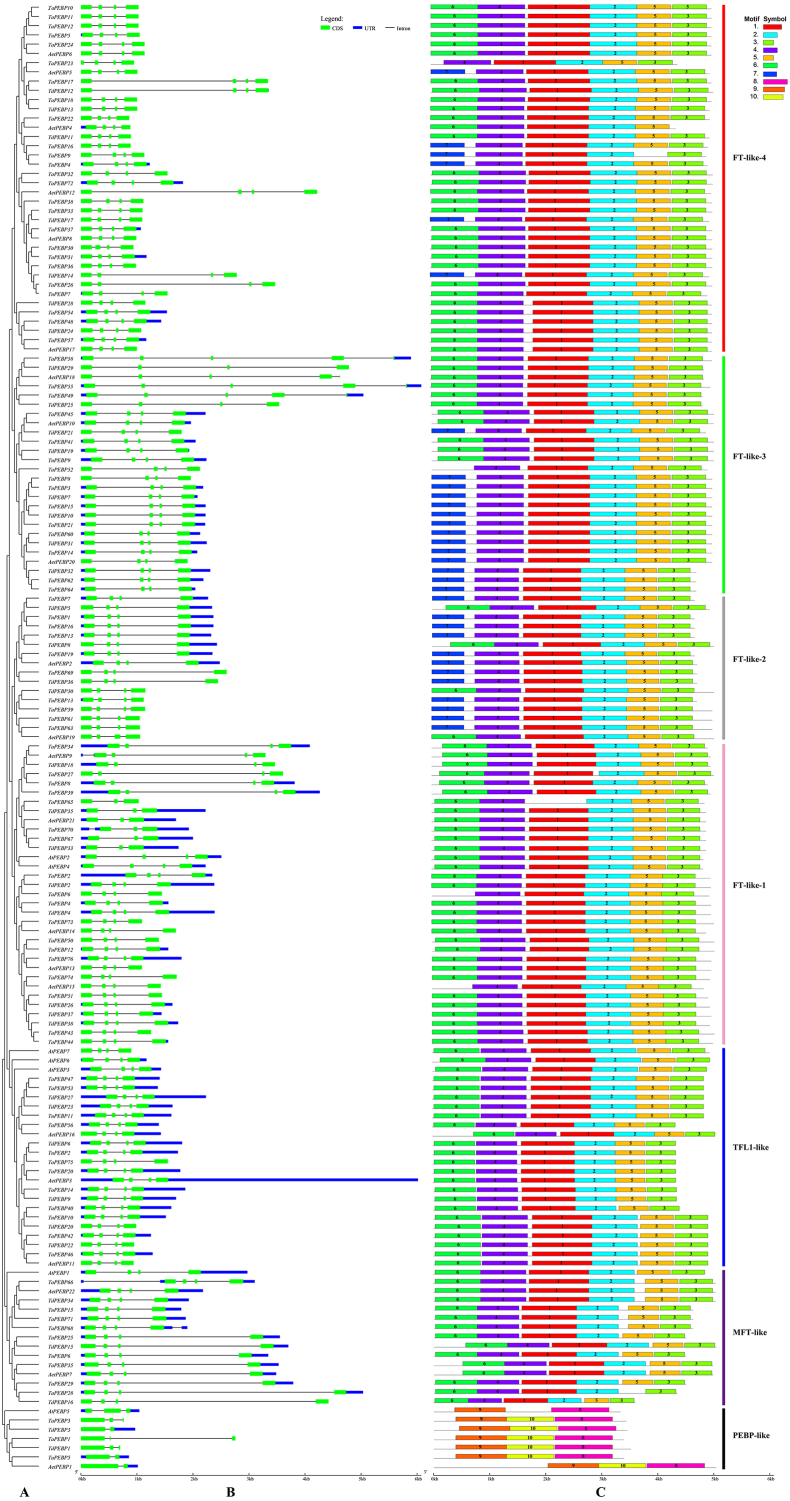
Phylogenetic relationships, gene structure and conserved motifs of the *PEBP* genes. (A) Rootless adjacent phylogenetic tree constructed using 152 wheat and its progenitors and 7 Arabidopsis PEBP protein sequences. (B) Exon/intron structure of the *PEBP* genes. Green boxes indicate exons, and black lines of the same length indicate introns. The untranslated regions (UTRs) of the *PEBP* genes are indicated with blue boxes. The length of the exon can be inferred from the ratio of the bottom. (C) Distribution of conserved motifs in the *PEBP* genes. Different colored boxes indicate different motifs, and the motif number in each gene has been indicated in the colored box. Vertical bars of different colors represent different subfamily classifications. See Table 1 for more information on motifs.

All of the identified 159 *PEBP* sequences, including seven *AtPEBP* sequences, were divided into four subfamilies, namely *PEBP-like*, *MFT-like*, *TFL-like* and *FT-like*, with 6, 14, 21, and 111 *PEBP* genes of wheat and its progenitors, respectively (Figs. 1, 2A). The *PEBP-like* subfamily was identified in this study for the first time. The *FT-like* subfamily members could be further divided into four classes: *FT- like-1*, *FT- like-2*, *FT- like-3* and *FT-like-4*.

### Gene structure and motif composition

The classic *PEBP* genes contain four exons (Danilevskaya et al., 2008). The 152 *PEBP* genes from common wheat and its progenitors identified in this study contained two to six exons (3 genes with 2 exons, 9 with 3 exons, 136 with 4 exons, 4 with 5 exons, and 6 with 6 exons) (Fig. 2B). A total of 10 conserved motifs (1–10) were identified in the predicted 159 *PEBP* genes, each comprising 20–50 aa (Fig. 2C, Table 1). PEBP proteins in the same subfamily had similar conserved motifs, which were distinct from those in PEBP proteins belonging to the other subfamilies. Except three genes (*AetPEBP4*, *TaPEBP9* and *TaPEBP65*), the *MFT-like*, *TFL-like* and *FT-like* subfamily genes contained motifs 1–5. Proteins in the FT-like-1 class, except TaPEBP65, contained seven motifs (1–7). On this basis, AetPEBP15 and TaPEBP65 lacked motifs 6 and 1, respectively, and only TaPEBP6 contained motif 7. All proteins in the FT-like-2 class contained six motifs (1–5 and 7). The FT-like-3 proteins contained seven motifs (1–7); The FT-like-4 proteins contained seven motifs (1–7). AetPEBP4, TaPEBP9 and TaPEBP23 contained six motifs, while the remaining proteins contained seven motifs. Proteins in the newly identified PEBP-like subfamily contained three motifs (8–10), which were different from those present in the other subfamilies. Motifs in the PEBP-like proteins of common wheat and its progenitor species were highly conserved; however, the AtPEBP5 protein lacked motif 10. To analyze sequence conservation of the proteins in the PEBP-like subfamily, we clustered the members of the PEBP-like subfamily in monocots (common wheat, *T. dicoccoides*, *Ae. tauschii*, *T. urartu*, *Hordeum vulgare*, *Oryza sativa*, *Sorghum bicolor*, *Zea mays* and Brachypodium) and dicots (Arabidopsis, cotton, soybean, tomato, cucumber and tobacco) (Table S2) and found that the PEBP-like subfamily was divided into two groups, indicating divergence of the PEBP-like subfamily genes between monocots and dicots. However, motif analysis showed that all PEBP-like subfamily proteins, except those in Arabidopsis and tomato, contained three motifs, and the amino acid sequence of the motifs was highly conserved (Fig. S1), indicating that PEBP-like proteins are conserved in plants.

**Table 1:** List of the putative motifs identified in PEBP proteins.

Motif	Width	Sites	Best possible match
Motif 1	38	151	LYTLVMVDPDAPSPSBPTLREYLHWLVTDIPGTTDASF
Motif 2	29	152	GTEVVPYESPKPTAGIHRFVFVLFRQLGR
Motif 3	21	151	LGLPVAAVYFNCQREGGCGGR
Motif 4	29	152	YGNREVTNGSELRPSAVANKPRVEIGGRD
Motif 5	21	150	QTVYAPGWRQNFNTRDFAECY
Motif 6	29	116	SRSRDPLVVGRVIGDVVDPFDPTVPLRVT
Motif 7	21	32	MSRDPLVVGRIVGDILDPFVK
Motif 8	50	7	NDWKQPGWRGPVPDSHGHRIQFRLYALDDVLSLGNKVTVDKVMEAIEGHV
Motif 9	44	7	LPRQYTLEGQGAKKDISPPLEWYGVPDGTRSLAVVVQDVDADER
Motif 10	41	6	VPWTHWVVVNISPEEKGLPEGFSGAGGNANAGGGDGGVQEG

### Chromosomal distribution and homology analysis of *PEBP* genes

**Figure 3:**
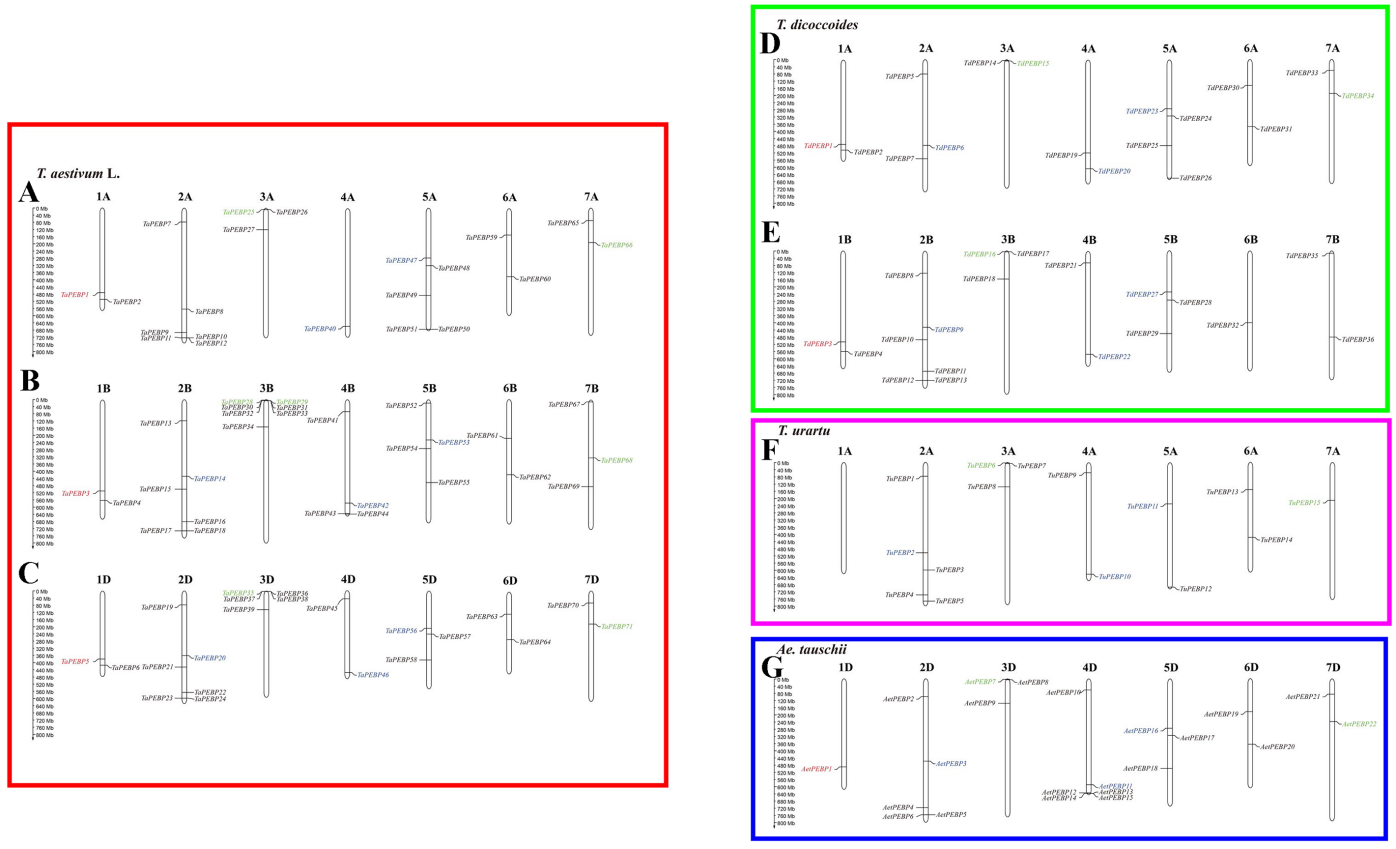
Chromosomal distribution of *PEBP* genes in wheat and its progenitor species. * PEBP-like*, *MFT-like*, *TFL1-like* and *FT-like* genes are indicated in red, green, blue and black, respectively. The names with the prefixes ‘*T. aestivum* L.’, ‘*T. dicoccoides*’, ‘*T. urartu*’, and ‘*Ae. tauschii*’, are for common wheat (A–C), *Triticum dicoccoides* (D–E), *Triticum urartu* (F) and *Aegilops tauschii* (G), respectively.

Out of 152 *PEBP* genes identified in wheat and its progenitors, 147 were mapped to chromosomes (Fig. 3). Group 2, 3 and 5 chromosomes harbored more *PEBP* genes than other chromosomes, with 37, 26 and 24 *PEBP* genes, respectively. In addition, group 1 chromosomes harbored the lowest number of *PEBP* genes, with only 11 in total. Chromosome 3B of common wheat harbored the highest number of *PEBP* genes (7), whereas chromosome 1A of *T. urartu* contained no *PEBP* genes.

Nine *TaPEBP* genes (*TaPEBP10*/*TaPEBP11*, *TaPEBP30*/*TaPEBP31*/*TaPEBP32*/*TaPEBP33*, *TaPEBP36*/*TaPEBP37*/*TaPEBP38*) clustered into four tandem repeat regions on chromosome 4D, and three *AetPEBP* genes (*AetPEBP13*/*AetPEBP14*/*AetPEBP15*) formed three clusters on chromosomes 3B, 3D and 4D of *Ae. tauschii*. Next, the Ka/Ks ratios of 10 tandem *PEBP* gene pairs were calculated. The Ka/Ks ratios of eight *PEBP* genes pairs were <1, and that of two gene pairs were >1 (Table 2), suggesting that most of the *PEBP* genes underwent intense purifying selection during evolution. In addition to tandem repeats, six pairs of segmental repeats consisting of eight genes were identified by MCScanX (Fig. S2). These data suggest that segmental and tandem duplication together led to the expansion of the *PEBP* family in wheat, with the latter being the main driving force.

**Table 2:** Ka/Ks analysis and estimated divergence time of *PEBP* genes pairs in common wheat and its progenitors.

Paralogous pairs	Origin	Ka	Ks	Ka/Ks	Divergence time (mya)
*TaPEBP10-TaPEBP11*	AABBDD	NA	NA	NA	NA
*TaPEBP30-TaPEBP31*	AABBDD	0.01	0.07	0.20	5.64
*TaPEBP31-TaPEBP32*	AABBDD	0.05	0.21	0.22	16.48
*TaPEBP32-TaPEBP33*	AABBDD	0.07	0.28	0.24	21.33
*TaPEBP36-TaPEBP37*	AABBDD	0.04	0.18	0.22	14.08
*TaPEBP36-TaPEBP38*	AABBDD	0.05	0.17	0.30	13.21
*TaPEBP37-TaPEBP38*	AABBDD	0.04	0.19	0.23	14.77
*AetPEBP13-AetPEBP14*	DD	1.05	0.86	1.23	65.82
*AetPEBP13-AetPEBP15*	DD	0.09	0.16	0.56	12.11
*AetPEBP14-AetPEBP15*	DD	1.01	0.98	1.03	75.39

**Figure 4:**
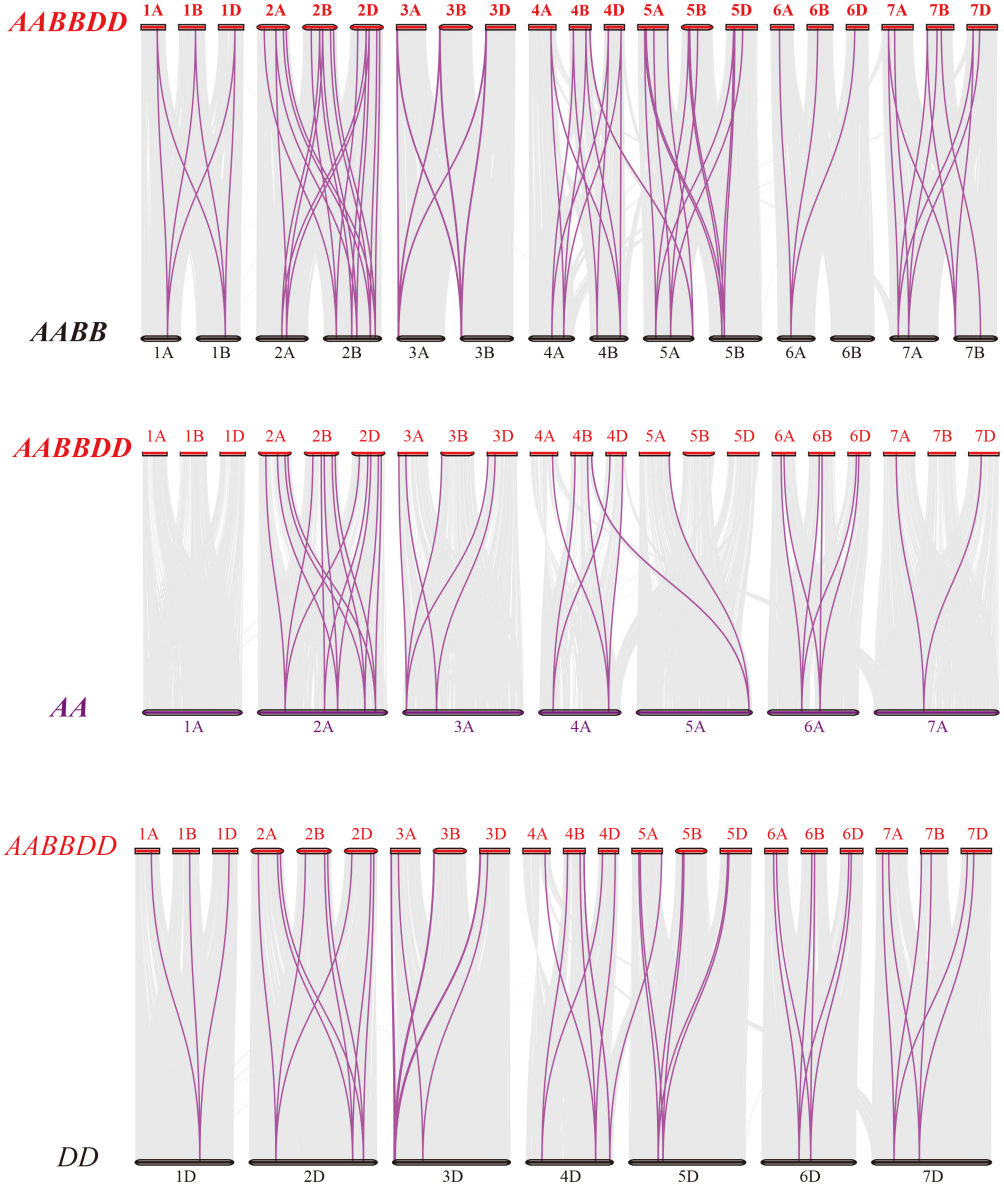
Synteny analysis of PEBP genes between wheat and its progenitors. Gray lines in the background indicate the collinear blocks within wheat and its progenitor species, while red lines highlight the syntenic *PEBP* gene pairs. Species with the AABBDD, AABB, AA and DD genomes indicate *Triticum aestivum* L., *T. dicoccoides*, *T. urartu* and *Aegilops tauschii*, respectively.

To further elucidate the phylogenetic mechanisms of the wheat *PEBP* family, we constructed a comparative map of common wheat, *T. dicoccoides*, *T. urartu* and *Ae. tauschii* (Fig. 4). A total of 51 *TaPEBP* genes were synonymous with *T. dicoccoides* genes, followed by *Ae. tauschii* (47) and *T. urartu* (36) (Table S3). It was found that 25 *TaPEBP* genes were also present in the three progenitor species, indicating that these genes likely played an important role in the evolution of the *PEBP* gene family. Interestingly, six *PEBP* genes identified as paralogs between common wheat and *T. dicoccoides* were not identified as paralogs between common wheat and *T. urartu*, suggesting that these paralogous pairs were formed after wheat tetraploidization. In addition, some genes (*TaPEBP20/21/43/44/58/61/62/68*) in wheat B and D subgenomes formed no homologous gene pairs or showed homologous relationships with other genes in other subgenomes. In addition, 15 wheat specific genes showed no collinearity with the PEBP genes in the donor species, suggesting that these genes are the result of gene loss, gene acquisition or chromosome translocation during wheat polyploidization.

### RNA-seq data of *TaPEBP* genes

Among the 76 *TaPEBP* genes, 37 showed no expression (TPM<1) in different tissues including root, stem, leaf, spike, embryo, endosperm, seed coat, stigma & ovary and anther, indicating that these genes might be pseudogenes or have a special spatiotemporal expression pattern, which was not detected in the transcriptome data. Of the 39 remaining *TaPEBP* genes, 36 were expressed in nine different tissues (TPM ≥ 1), and three genes (*TaPEBP5*, *TaPEBP27* and *TaPEBP39*) showed structural expression (TPM >1) (Fig. S3, Table S4). The *TaPEBP* gene family members were expressed in different tissues and showed different expression patterns. Expression patterns of genes were similar within subfamilies. Some genes showed preferential expression (TPM >10) in different tissues, such as *TFL1-like* genes (*TaPEBP47*/53/56) in the roots, *FT-like-1* genes (*TaPEBP27/39/67/70/76*) and *TFL1-like* genes (*TaPEBP40* and *TaPEBP53*) in the stem, *FT-like-1* gene (*TaPEBP27*) and *PEBP-like* genes (*TaPEBP1*/3/5) in the leaf, *MFT-like* genes (*TaPEBP66*/68/71) and *PEBP- like* genes (*TaPEBP1*/3/5) in the endosperm, *MFT-like* genes (*TaPEBP66*/68), *FT-like-1* genes (*TaPEBP27/34/39*) and *PEBP-like* genes (*TaPEBP1*/3) in the seed coat. Additionally, *PEBP-like* genes (*TaPEBP1*/3/5), *MFT-like* genes (*TaPEBP25*/29/35) and *TFL1-like* gene (*TaPEBP35*) were highly expressed in the embryo, whereas the *TFL-like* gene (*TaPEBP20*), *FT-like-2* gene (*TaPEBP27*), and *PEBP-like* gene (*TaPEBP5*) were highly expressed in the stigma, ovary, spike and anther. All *PEBP-like* genes (*TaPEBP1*/3/5) and *MFT-like* genes (*TaPEBP25/28/29/35/66/68/71*) showed similar expression patterns during grain development, reaching a peak at 20 DPA, followed by a gradual decline (Fig. S3, Table S4).

**Figure 5:**
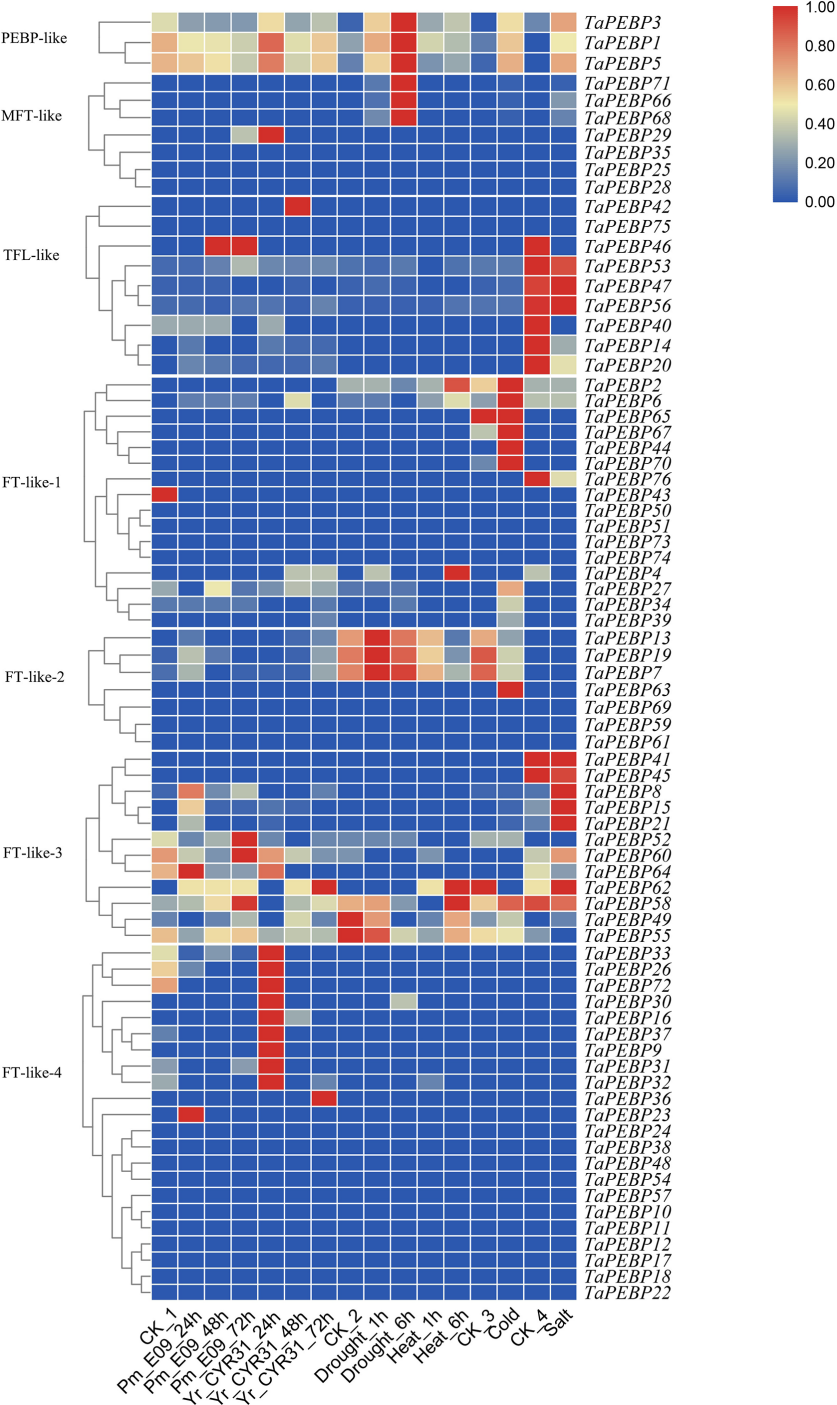
Expression patterns of *TaPEBP* genes under various stress treatments. The heat map generated by TBtools shows the cluster map of *TaPEBP* genes under powdery mildew, stripe rust, drought, heat, cold and salt treatments. The color gradient (red/white/blue) indicates the gene expression level (from high to low). CK_1_ is the control of powdery mildew and stripe rust; CK_2_ is the control of drought and heat treatments; CK_3_ is the control of cold treatment; CK_4_ is the control of salt stress treatment. Each subfamily was clustered separately.

We also analyzed the expression profiles of *PEBP* genes in wheat under biotic and abiotic stresses (Fig. 5). Of the 76 *TaPEBP* genes, 25 were expressed under one or more stress treatments (TPM >1), and 10 of these genes showed TPM >10 under different stress treatments (Table S4). Most of the *TaPEBP* genes were not highly expressed. Only *PEBP-like* and *FT-like-3* genes responded significantly to salt stress, while *FT-like-2* and *FT-like-3* genes responded to drought and heat stresses. The expression of *FT-like-3* and *FT-like-4* genes changed in response to infection by powdery mildew and stripe rust pathogens. The newly identified *PEBP-like* genes (*TaPEBP1*/3/5) were highly responsive to both biotic and abiotic stress treatments (Fig. 5, Table S4).

**Figure 6:**
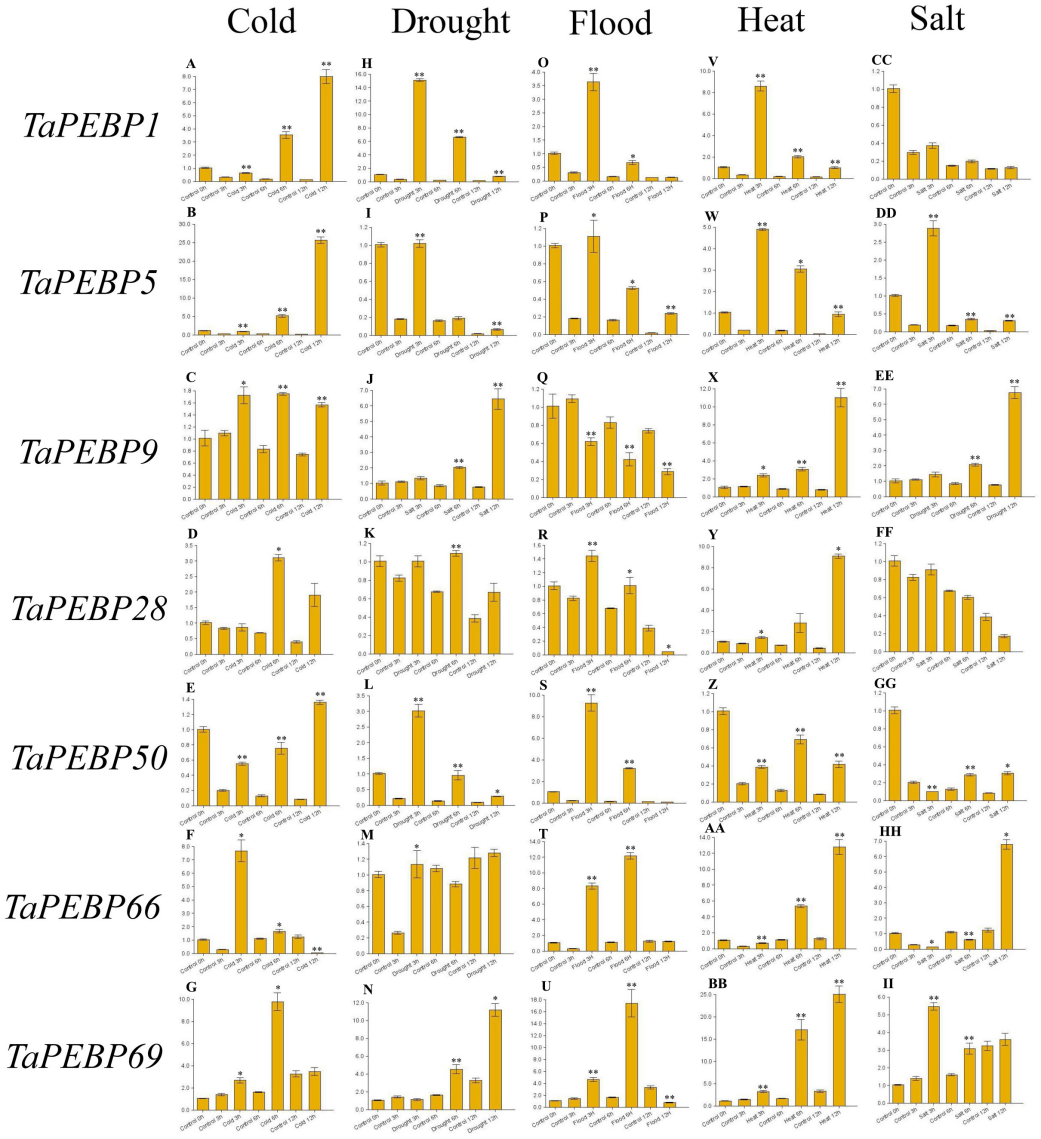
Expression levels of seven PEBP genes in wheat under cold (A–G), drought (H–N), flood (O–U), heat (V-BB), and salt (CC-II) stresses validated by qRT-PCR. Seeds were treated with cold (four), drought (20% PEG6000), flood, heat (45 °C) and salt (200 mM NaCl), and embryos were sampled at 0, 3, 6 and 12 h. Data represent mean ± standard error (SE) of three biological replicates. Statistically significant differences between the control and treatment groups are indicated using asterisks (* *p* < 0.05, ** *p* < 0.01; independent Student’s *t*-test).

### Expression of *TaPEBP* genes under adverse conditions during seed germination

To study the role of *PEBP* family genes during seed germination, the expression profiles of seven genes were examined under cold, drought, flood, heat and salt stress treatments by qRT-PCR (Fig. 6, Table S4). Except for *TaPEBP1* and *TaPEBP28* that showed no expression difference under salt stress, the other genes show differential expression under other treatments. The response patterns of the seven genes to salt stress varied, with *TaPEBP5* and *TaPEBP50* showed significant expression differences at different time points. Under cold and heat stresses, all seven genes were up-regulated, with the expression patterns of *TaPEBP1* and *TaPEBP5* were similar in cold and hot conditions, whereas those of *TaPEBP9* and *TaPEBP66* were similar under heat treatment. Under drought stress, all genes, except *TaPEBP66*, were up-regulated to varying degrees. The expression patterns of *TaPEBP1*, *TaPEBP5* and *TaPEBP50* were the same but opposite to those of *TaPEBP9* and *TaPEBP69*. In the flood treatment, except *TaPEBP9*, all the other genes were up-regulated, the expression of *TaPEBP66* and *TaPEBP69* was dynamic, increasing until 6 h and then gradually decreasing. The same TaPEBP gene showed different response to different stress treatments, indicating that the same TaPEBP gene might play different roles under different stresses. In addition to salt treatment, the expression of *TaPEBP1* and *TaPEBP5* not only changed significantly, but also showed similar trends, indicating that the members of the PEBP-like subfamily were conservative in function.

## Discussion

Growing evidence indicates that functional units of PEBP proteins have an ancient common origin, and are significantly related to the growth and development of plants and their seasonal adaptability (Pnueli et al., 1998). Although the importance of the *PEBP* gene family in cereals has been published previously, wheat *PEBP* genes were mainly analyzed from public EST data, which was insufficient because of the lack of whole genome sequence of wheat (Chardon & Damerval, 2005). In this study, we address this gap by identifying the entire *PEBP* gene family in wheat and its progenitor species using whole genome sequences. We analyzed the complete gene and protein structures, evolutionary relationships with Arabidopsis and spatiotemporal gene expression patterns. We identified 76, 38, 16 and 22 *PEBP* genes in common wheat, *T. dicoccoides*, *T. urartu* and *Ae. tauschii* by genome-wide searches and protein sequence alignments. Phylogenetic analysis revealed that the *PEBP* genes of common wheat and its progenitor species, unlike those of other plant species, were divided into four subfamilies, PEBP-like, TFL1-like, MFT-like and FT-like, which were different from the three main subfamilies identified by predecessors (Kardailsky et al., 1999). In addition, we identified the *PEBP-like* gene subfamily in various monocotyledons, including *H. vulgare*, *O. sativa* ssp. *japonica*, *S. bicolor*, *Z. mays* and *B. distachyon*, and dicotyledons such as *A. thaliana*, *Gossypium raimondii*, *G. max*, *S. lycopersicum*, *C. sativus*, and *N. attenuata*. Cluster and motif analyses revealed the *PEBP* genes of common wheat, *T. dicoccoides*, *T. urartu*, *Ae. tauschii* and the above mentioned species. Although the *PEBP-like* subfamily genes showed differences between monocotyledons and dicotyledons, the sequences were highly conserved, suggesting that the *PEBP-like* subfamily performed important functions during evolution. The *PEBP-like* subfamily was identified in this study for the first time using the HMM model, indicating that the HMM model provides powerful support for the integrity and accuracy of gene families.

The *PEBP* family is a large taxon in Triticeae crops similar to that in other plant species, with six genes in Arabidopsis, 24 in maize, 23 in soybean, 19 in rice, 12 in tomato, 19 in sorghum, and 20 in *Setaria italica* (Kardailsky et al., 1999; Liu et al., 2016; Danilevskaya et al., 2008; Wang et al., 2015). In this study, we mapped 71 *TaPEBP*, 36 *TdPEBP*, 15 *TuPEBP* and 22 *AetPEBP* genes to their respective chromosomes (Fig. 3). Although hexaploid wheat is formed as a result of two hybridization events, the number of *PEBP* genes in each wheat subgenome is not the same as that in the corresponding progenitor species genomes. Hexaploid wheat is derived from three diploid ancestors through two interspecific crosses. The first hybridization event generated the heterotetraploid species, *T. dicoccoides*. The number of *PEBP* genes in the A subgenome of *T. dicoccoides* (17) increased by two compared with *T. urartu* (15). The second hybridization event formed the heterohexaploid, in which the number of *PEBP* genes in the A and B subgenomes increased by four and nine, respectively. In addition, compared with *Ae. tauschii*, the number of *PEBP* genes in the D subgenome of common wheat didn’t change, but the number of PEBP genes on 1D, 2D and 3D increased, whereas those on 4D decreased. Doubling of the genome during the formation of hexaploid wheat may be the reason for the increase or loss of *PEBP* genes. A total of 13 *TaPEBP* duplicated genes were detected in common wheat, including six pairs of segmentally duplicated genes and seven tandemly duplicated genes (Fig. 3, Table 2, Fig. S2), indicating that both tandem and segmental duplications contributed to the evolution and increase of *TaPEBP* genes in common wheat, however, these tandem repeat genes are not expressed in different tissues and stress treatments, which might be caused by the spatiotemporal expression of genes and some of the specific period or process were not involved in our study (Fig. 5, Fig. S3, Table S4).

The ratio of *TFL1-like*: *MFT-like*: *FT-like* genes was 4:2:13 and 6:3:15 in rice and maize, respectively (Chardon & Damerval, 2005; Danilevskaya et al., 2008), but 2:3:11, 3:6:27, 2:3:16 and 7:9:56 in *T. urartu*, *T. dicoccoides*, *Ae. tauschii* and common wheat, respectively, indicating that the *FT-like* genes in common wheat and its progenitor species increased in numbers compared with rice and maize. An ancient whole genome duplication (WGD) event has been reported in the ancestors of existing angiosperms, which resulted in duplicate copies of each gene including flowering regulatory genes (Jiao et al., 2011; Jiao et al., 2014). In the *PEBP* subfamily, new gene duplication or genome duplication is often detected in angiosperms and gymnosperms. All monocotyledons underwent ancestral replication events during early evolution, which coincided with the new discovery of ancient WGD events before the divergence between Gramineae and Archaea (Jiao et al., 2014). Most dicot species have no more than 10 *PEBP* genes. Compared with similar species, these species have been shown to experience additional WGD events. For example, in Brassicales, *Brassica* experienced an additional recent genome-wide triplication (WGT) event compared with Arabidopsis (Liu et al., 2016). Similarly, WGDs may also promote the diversity of *PEBP* genes in soybean and apple (*Malus domestica*) (Liu et al., 2016; Wang et al., 2015). Therefore, in combination with the divergence time of tandem repeat genes, multiple WGD events during evolution are undoubtedly responsible for the large number of *PEBP* genes in common wheat.

The tissue/organ-specific expression pattern usually reflects the corresponding biological function of a gene. The RNA-seq data of different wheat tissues showed that *FT-like-1* genes (*TaPEBP27/39/67/70/76*) and *TFL1-like* genes (*TaPEBP40* and *TaPEBP53*) were highly expressed in the stem, MFT-like genes (*TaPEBP66*/68/71) and *PEBP-like* genes (*TaPEBP1*/3/5) in the endosperm, *TaPEBP25*, *TaPEBP29* and *TaPEBP35* in the embryo, *MFT-like* genes (*TaPEBP66*/68), *FT-like-1* genes (*TaPEBP27/34/39*) and *PEBP-like* genes (*TaPEBP1*/3) in the seed coat and *PEBP-like* gene (*TaPEBP5*) in the anther. Some *PEBP* genes are not expressed in any tissues, it is possible that these genes have tissue-specific and time- specific expression pattern and the current RNA-seq data didn’t reveal the expression pattern of these special genes/tissue/time. These results suggest that *PEBP* genes play different roles at different stages during plant growth and development (Karlgren et al., 2011; Kikuchi et al., 2009).

The expression profiles of *TaPEBP* genes under biotic and abiotic stresses were also investigated (Fig. 5). The expression of *FT-like-3* and *FT-like-4* genes changed in response to infection by powdery mildew and stripe rust pathogens. Genes in *FT-like-2* and *FT-like-3* classes responded to drought and heat. PEBP-like genes (*TaPEBP1*, *TaPEBP3* and *TaPEBP5*) were up-regulated in response to stripe rust, drought, heat and cold treatments, and these results were confirmed by qRT-PCR, indicating that the *PEBP* family plays an important role in biotic and abiotic stress responses. Our findings are consistent with those obtained in previous studies, including genes such as *TaFT3* (*TaPEBP2*), *VRN3* (*TaPEBP67*) and *TaPHS1* (*TaPEBP25*) (Fang et al., 2019; Hamoud et al., 2016; Ryu, Park & Seo, 2011; Liu et al., 2013). In this study, some of the results of qRT-PCR and RNA-seq are inconsistent, which may be caused by the inconsistency of materials. However, we still found some genes showed consistent expression among different species, such as *TaPEBP25,* which was consistent with that of Arabidopsis (*AtMFT*), and *TaPEBP67*, which was consistent with that of *HvFT1* and *HvFT2* in barley and *Hd3a in* rice, suggesting the functional conservation of PEBP gene in the evolution process (Xi et al., 2010; Liu et al., 2013; Kikuchi et al., 2009; Kojima et al., 2002). The newly discovered *PEBP-like* genes were not only expressed in response to abiotic and abiotic stresses such as cold, drought, salt and stripe rust, but were also highly expressed in leaves and grains, and reaching a peak at 20 DPA (Fig. S3), suggesting that the *PEBP-like* subfamily plays an important role in disease resistance, stress tolerance and grain development in wheat, although the specific functions of these genes need to be further investigated.

## Conclusion

Our results provide new evidence for further understanding the structure, evolution and function of *PEBP* family genes in common wheat and its progenitor species. Furthermore, the results of this study provide a basis for further understanding the role of *TaPEBP* genes in the vegetative-to-reproductive phase transition and grain dormancy as well as seed germination under adverse conditions.

##  Supplemental Information

10.7717/peerj.10483/supp-1 Supplemental Information 1 List of primers used for *TaPEBP.* gene expression analysis

10.7717/peerj.10483/supp-2 Supplemental Information 2 Identification of PEBP protein in wheat, its progenitors and *Arabidopsis*

10.7717/peerj.10483/supp-3 Supplemental Information 3 Collinearity analysis of PEBP genes between wheat and its progenitor species

10.7717/peerj.10483/supp-4 Supplemental Information 4 The expression data of PEBP genes in different tissues, multiple stress treatments and RT-qPCR

10.7717/peerj.10483/supp-5 Supplemental Information 5 Cluster and motif analyses of *PEBP- like* genes in monocotyledons and dicotyledons Different colored boxes indicate different motifs, and motifs in each gene are indicated in the colored boxes. In addition, species with black gene IDs are monocotyledons, and those with red gene IDs are dicotyledons.

10.7717/peerj.10483/supp-6 Supplemental Information 6 Gene duplication identified in wheat. Seven chromosomes in each subgenome of wheat (A, B and D) are indicated in different colors. Duplicated gene pairs are connected with lines of the corresponding color.

10.7717/peerj.10483/supp-7 Supplemental Information 7 Expression analysis of *TaPEBP* genes in nine different tissues and in grains at different developmental stages Heatmap generated using TBtools shows the cluster map of *TaPEBP* genes in roots, stem, leaf, spike, embryo, endosperm, seed coat, stigma & ovary, anther and grain (at 10, 20 and 30 days post anthesis [DPA]). The color gradient (red/white/blue) indicates the gene expression level (from high to low). Each subfamily formed a separate cluster.

## Additional Information and Declarations

### Data Availability

The following information was supplied regarding data availability: Raw data, the PEBP gene identification results and gene expression data, are available in the Supplemental Files.
