# Supplementary figures and images for: Genome-wide member identification, phylogeny and expression analysis of PEBP gene family in wheat and its progenitors
Source: PeerJ. 2020 Dec 15;8:e10483. doi: 10.7717/peerj.10483 (PMC7747686; doi:10.7717/peerj.10483)

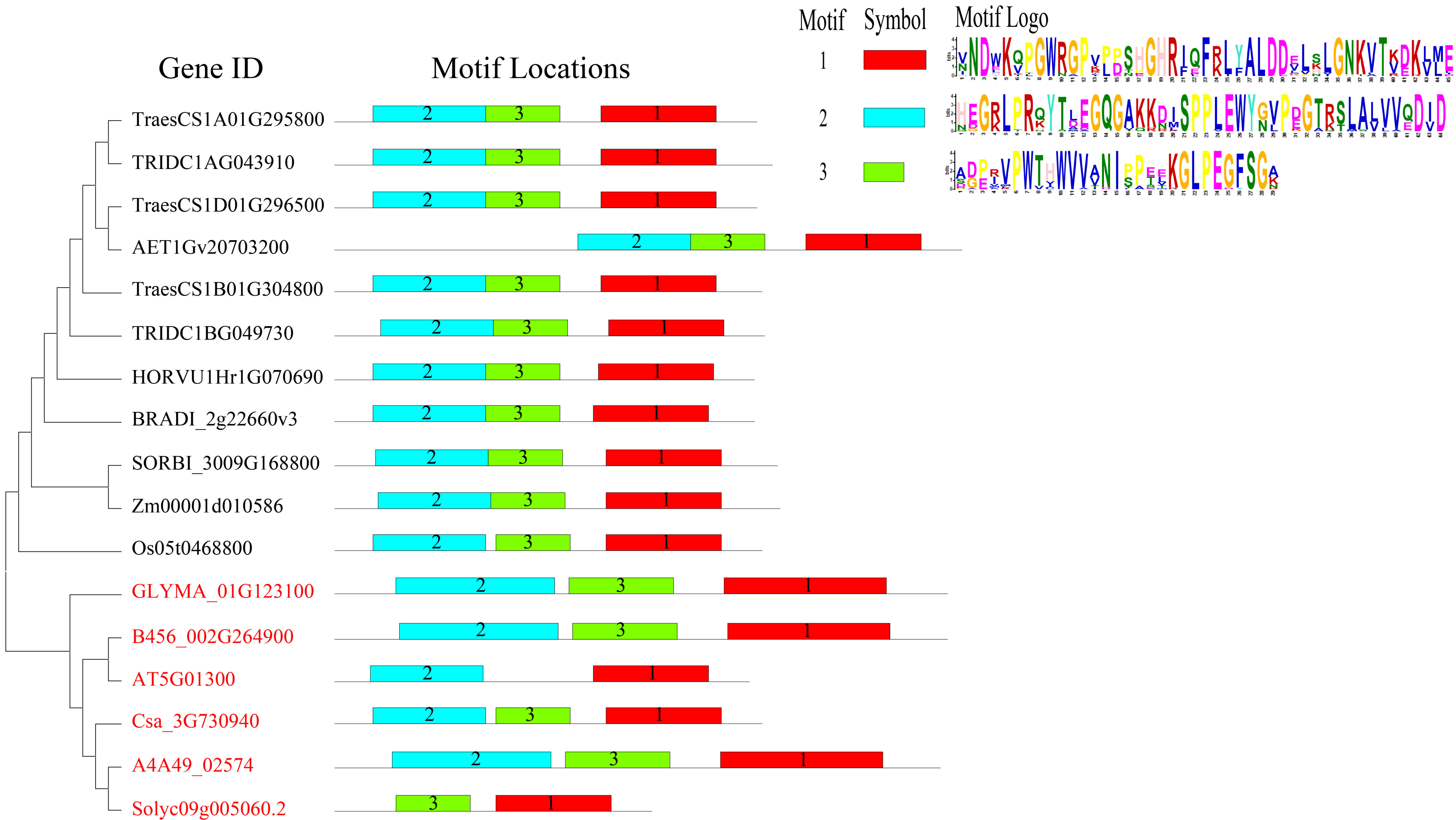

Supplement: Supplemental Information 5 — Different colored boxes indicate different motifs, and motifs in each gene are indicated in the colored boxes. In addition, species with black gene IDs are monocotyledons, and those with red gene IDs are dicotyledons. [file peerj-08-10483-s005.pdf]

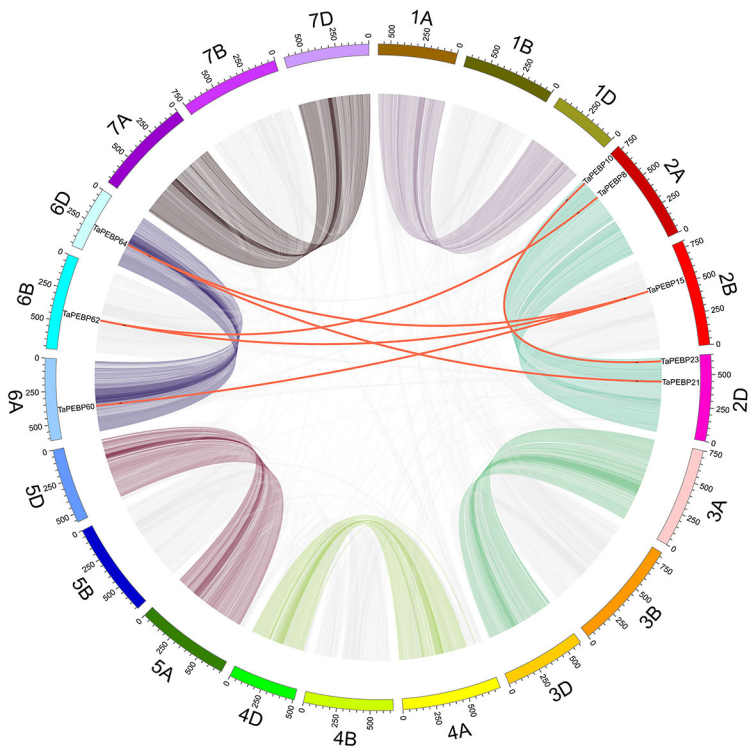

Supplement: Supplemental Information 6 — Seven chromosomes in each subgenome of wheat (A, B and D) are indicated in different colors. Duplicated gene pairs are connected with lines of the corresponding color. [file peerj-08-10483-s006.pdf]

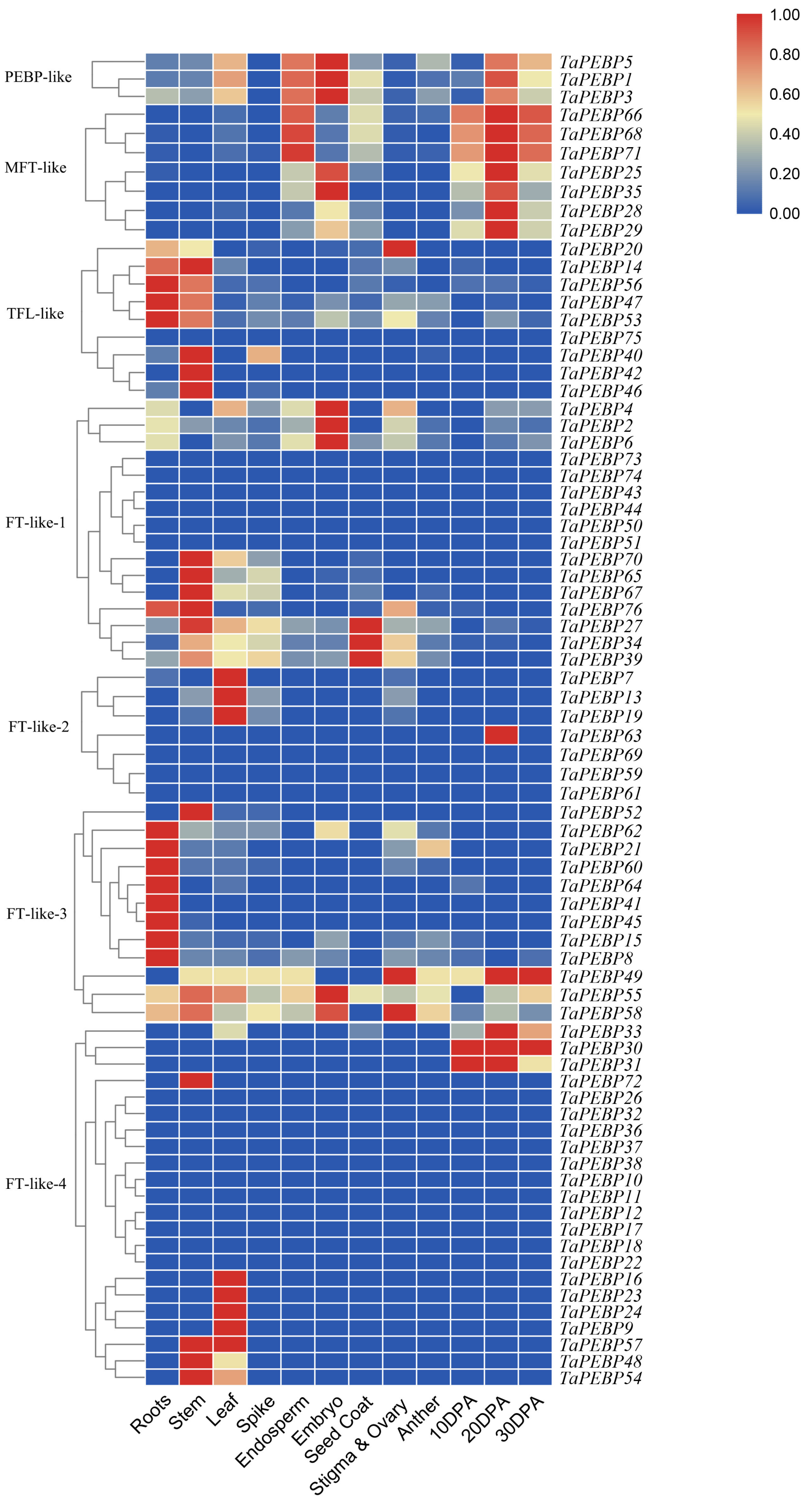

Supplement: Supplemental Information 7 — Heatmap generated using TBtools shows the cluster map of TaPEBP genes in roots, stem, leaf, spike, embryo, endosperm, seed coat, stigma & ovary, anther and grain (at 10, 20 and 30 days post anthesis [DPA]). The color gradient (red/white/blue) indicates the gene expression level (from high to low). Each subfamily formed a separate cluster. [file peerj-08-10483-s007.pdf]
